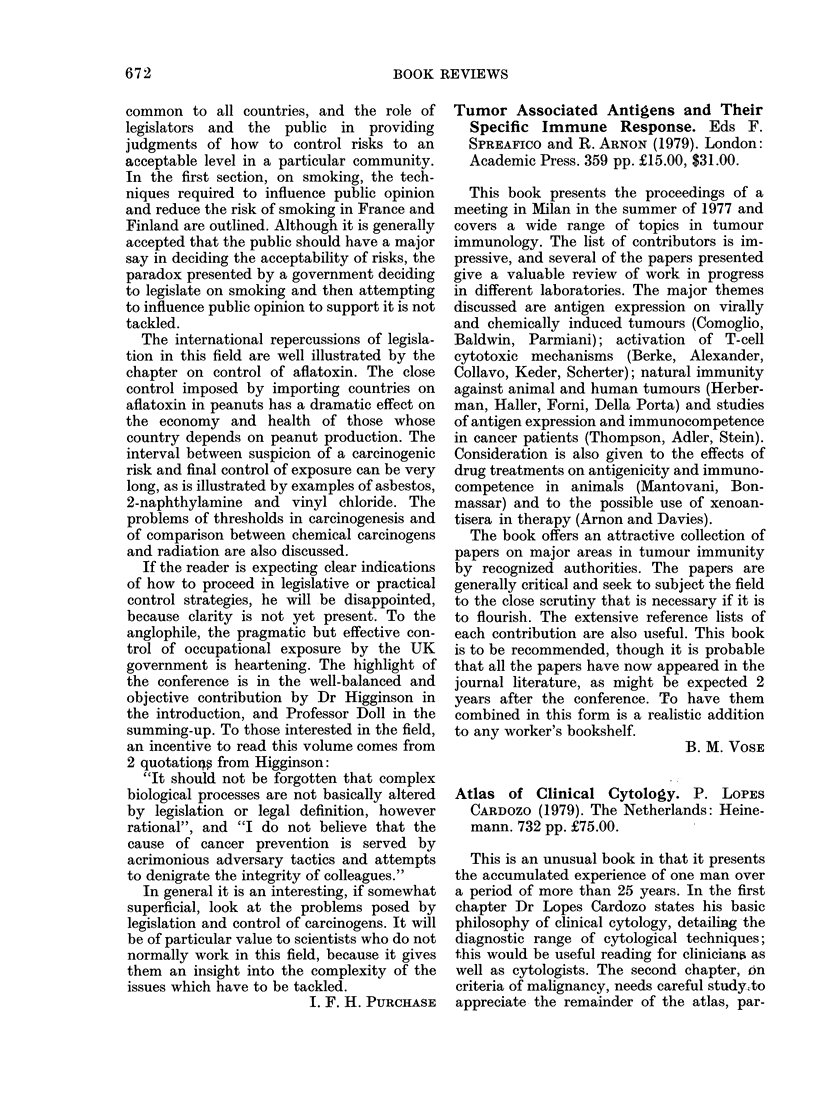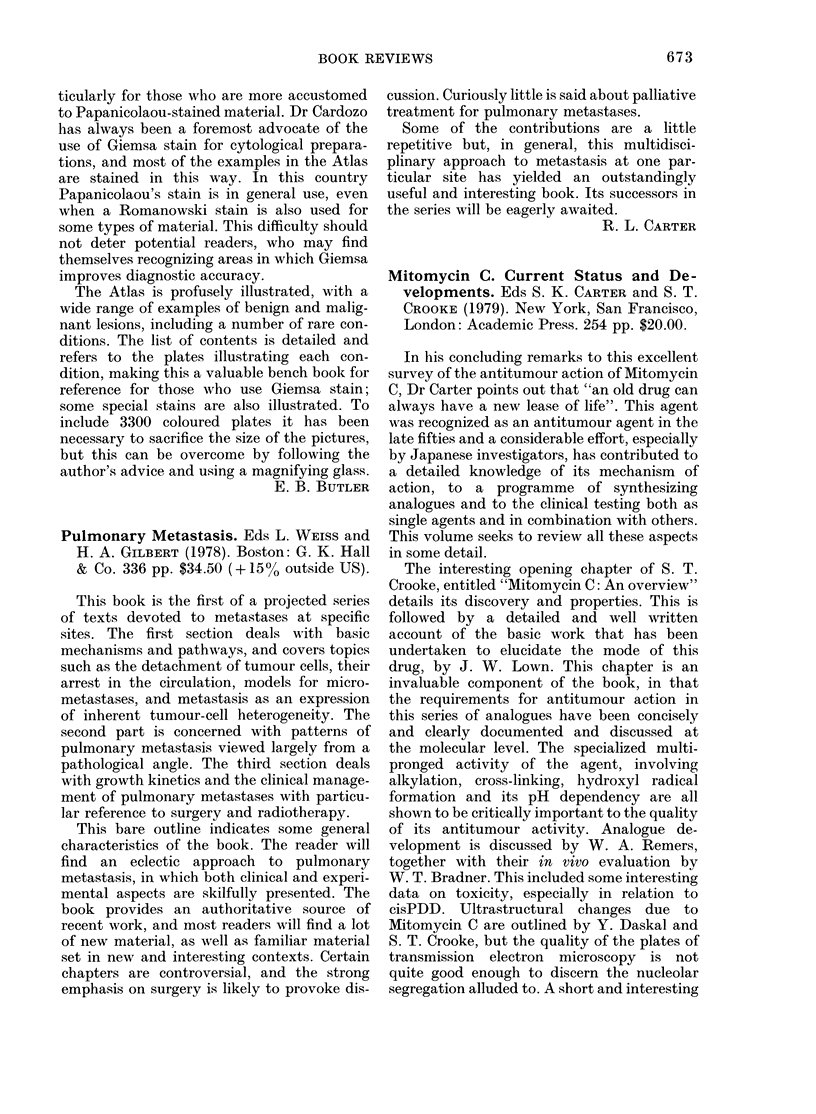# Atlas of Clinical Cytology

**Published:** 1980-04

**Authors:** E. B. Butler


**Atlas of Clinical Cytology.** P. LOPES CARDOZO (1979). The Netherlands: Heinemann. 732 pp. £75.00.

This is an unusual book in that it presents the accumulated experience of one man over a period of more than 25 years. In the first chapter Dr Lopes Cardozo states his basic philosophy of clinical cytology, detailing the diagnostic range of cytological techniques; this would be useful reading for clinicians as well as cytologists. The second chapter, on criteria of malignancy, needs careful study to appreciate the remainder of the atlas, particularly for those who are more accustomed to Papanicolaou-stained material. Dr Cardozo has always been a foremost advocate of the use of Giemsa stain for cytological preparations, and most of the examples in the Atlas are stained in this way. In this country Papanicolaou's stain is in general use, even when a Romanowski stain is also used for some types of material. This difficulty should not deter potential readers, who may find themselves recognizing areas in which Giemsa improves diagnostic accuracy.

The Atlas is profusely illustrated, with a wide range of examples of benign and malignant lesions, including a number of rare conditions. The list of contents is detailed and refers to the plates illustrating each condition, making this a valuable bench book for reference for those who use Giemsa stain; some special stains are also illustrated. To include 3300 coloured plates it has been necessary to sacrifice the size of the pictures, but this can be overcome by following the author's advice and using a magnifying glass.

E. B. BUTLER